# Self-Assembled TLR7/8 Agonist-Mannose Conjugate as An Effective Vaccine Adjuvant for SARS-CoV-2 RBD Trimer

**DOI:** 10.3390/polym14245466

**Published:** 2022-12-13

**Authors:** Changcai Teng, Xiongyan Meng, Yeqin Hu, Hongzhao Mao, Huiting Li, Jing Yang, Tiantian Sun, Shuai Meng, Chengli Zong

**Affiliations:** 1 Key Laboratory of Tropical Biological Resources of Ministry of Education, School of Pharmaceutical Sciences, Hainan University, Haikou 570228, China; 2 MAXVAX Bio-tech Co., Ltd., Chengdu 610200, China

**Keywords:** adjuvants, mannose, TLR7/8 agonist, immune responses, cytokines, antibody

## Abstract

Small synthetic TLR7/8-agonists can be used as vaccine adjuvants to enhance cell and humoral-mediated immune responses to specific antigens. Despite their potency, after local injection they can be dispersed to undesired body parts causing high reactogenicity, limiting their clinical applications. Here we describe a vaccination strategy that employs the covalent conjugate of a mannose and TLR7/8 agonist as a vaccine adjuvant to take advantage of mannose binding C-type lectins on dendritic cells to enhance the vaccine’s immunogenicity. The mannose-TLR7/8 agonist conjugate can self-assemble into nanoparticles with the hydrophilic mannose on the outside and hydrophobic TLR7/8 agonist inside. Although its ability to stimulate HEK-Blue^TM^ hTLR7/8 cells dropped, it can efficiently stimulate mouse bone marrow-derived dendritic cells as indicated by the up-regulation of CD80 and CD86, and higher cytokine expression levels of TNF-α, IL6, and IL-12p70 than the native TLR7/8 agonist. In vivo, vaccination using the SARS-CoV-2 RBD trimer as the antigen and the conjugate as the adjuvant induced a significantly higher amount of IgG2a. These results suggest that the mannose-TLR7/8-agonist conjugate can be used as an effective vaccine adjuvant.

## 1. Introduction

Toll-like receptors (TLRs) are typical pattern recognition receptors (PRRs). They can recognize a broad spectrum of pathogen-associated molecules, such as lipoproteins (TLR2), double-stranded ribonucleic acid (RNA, TLR3), lipopolysaccharides (TLR4), flagellin (TLR5), single-stranded viral RNA (TLR7/8), and unmethylated CpG (consisting of a central unmethylated CG dinucleotide plus flanking regions) DNA of bacteria and viruses (TLR9). After recognition of their ligand, all TLRs signal through myeloid differentiation primary response gene 88 (MyD88) or TIR-domain-containing adaptor molecule 1 (TRIF), leading to the activation of nuclear factor kappa B (NF-kB) or interferon regulatory factor 3 (IRF3), and consequently the activation of many genes involved in innate immunity. They have attracted huge interest as novel vaccine adjuvants. Detoxified lipid A: monophosphate lipid A (MPLA), is among the first of a new generation of TLR agonists used as vaccine adjuvants on a mass scale in human populations [1]. However, MPLA production remains a huge challenge due to the difficult quality control in lipid fermentation of source bacteria or total chemical synthesis. Although the natural ligand for TLR7/8 are single-stranded RNA (ssRNA), synthetic imidazoquinoline agonists bind well with the TLR7/8 receptor, and are being investigated as vaccine adjuvants. Imiquimod (R848) was approved by Food and Drug Administration (FDA) for external genital warts, superficial basal cell carcinoma, and actinic keratosis. However, early TLR7/8 agonists like R848 suffered from diffusion to undesired sites, leading to high reactogenicity and limiting their clinical applications [2]. Novel TLR7/8 agonists without systemic immune responses are of great interest. Recently, several groups have developed lipidated or alum-adsorbed TLR7/8 ligands to overcome the rapid systemic distribution and toxicity noted with the previous compounds [3,4,5,6].

The mannose receptor (MR) is a member of the C-type lectin receptor (CLR) family, expressed at a high level on dendritic cells (DCs) and involved in MR-mediated endocytosis and phagocytosis [3]. It is an important pathway for antigen uptake and delivery to major histocompatibility complex (MHC) class II molecules [4,5]. Various mannose structures have been conjugated with many types of antigens to improve vaccine efficacy [6]. For example, Hubbell et al. demonstrated that the antigens reversibly conjugated to a polymeric mannose and TLR7/8 agonist induced protective humoral and cellular immunity [7]. We found the covalent modification of a polymer with the agonist and mannose is non-trivial; the loading level is usually very low. Although many groups have demonstrated that a covalently linked antigen and adjuvant can significantly boost their immune response [8,9], others have argued that the covalent linker might generate undesirable antigenic effects [10,11,12,13]. Furthermore, the covalent conjugation of antigen and adjuvant may damper the intracellular process of antigens into peptides and the loading of peptides onto major histocompatibility complex molecules for presentation to T cells.

The novel coronavirus disease, caused by SARS-CoV-2 (COVID-19), has become the most influential epidemic in recent years [14,15]. COVID-19 vaccines are effective at protecting people from getting seriously ill, being hospitalized, and dying. New vaccines or vaccination strategies that are safer and more effective are still needed to cope with the continuous evolution of the virus. The receptor-binding domain (RBD) of the spike protein mediates viral entry into host cells by first binding to a host receptor and has been proven as an effective vaccine antigen [16].

Here, we describe a vaccine strategy that leverages the ability of mannose binding C-type lectins on DCs for targeted TLR7/8 agonist adjuvant delivery. The mannose-TLR7/8 agonist conjugate can self-assemble into nanoparticles. The polymeric mannose structure displayed on the nanostructure are designed to enhance the DC recognition by binding MRs. The activated DC can take in the co-injected antigen to cascade the following humoral and cellular immune response. We demonstrate the conjugate can effectively activate bone marrow-derived dendritic cell (BMDC). The conjugate can elicit high titers of IgG antibodies against the receptor-binding domain (RBD) trimer.

## 2. Materials and Methods

### 2.1. TLR Reporter Assay

HEK-Blue^TM^ hTLR7 and hTLR8 cells (InvivoGen, Hongkong, China) were cultured in DMEM supplemented with 10% Fetal Bovine Serum (FBS), Penicillin-Streptomycin (Beyotime, Shanghai, China), blasticidin (InvivoGen), zeocin (InvivoGen), and normocin (InvivoGen). These reporter cells (InvivoGen) were harvested and mixed with HEK-Blue™ Detection (InvivoGen); the mixture was distributed into 96-well plates at 2.5 × 10^4^ cells/well, then treated with dimethyl sulfoxide (DMSO), TLR7/8 (Compound 2) agonist, or Mannose-TLR7/8 (Compound **3**) at different concentrations (0.006 μM, 0.01 μM, 0.03 μM, 0.06 μM, 0.1 μM, 0.3 μM, 0.6 μM, 1 μM, 3 μM, 6 μM, and 10 μM) for 14 h. We determined secreted embryonic alkaline phosphatase (SEAP) activity by reading the optical density (OD) at 630 nm on a SpectraMax-M2 (Molecular Devices, San Jose, CA, USA) plate reader.

### 2.2. In Vitro BMDCs Assay and Cytokine Assay

BMDCs were isolated from hind limb bones of 6–8 weeks old Balb/c mice (Guangzhou Yancheng Biotech Company, Guangzhou, China). The red blood cells were lysed and the remaining cells were centrifuged at 450 g for 5 min. Then, 2 × 10^6^ BMDCs were seeded to a 6-well plate and cultured with Roswell Park Memorial Institute (RPMI)1640 supplemented with 10% FBS, 20 ng/mL Granulocyte-Macrophage Colony Stimulating Factor (GM-CSF, Sino Biological, Peking, China), and 10 ng/mL Interleukin 4 (IL-4, Sino Biological) and incubated at 37 °C. On day 7, the non-adherent cells were added to the wells of a 24-well plate (1.5 × 10^6^ BMDC/well). DMSO, Compound **2**, or Compound **3** (3 μM) were added to the cells and incubated overnight. After 24 h, cells were harvested and supernatants were collected. The cells were washed and stained with fluorophore labeled mAb to measure co-stimulatory molecule expression (CD11c+-FICT, CD86-APC, Biolegend, CD80-PE, eBioscience, Hongkong, China) by flow cytometry. The amount of Interleukin 12p70 (IL-12p70), Interleukin 6 (IL6), interferon gama (INF-γ), and tumor necrosis factor alpha (TNF-α) in the supernatants were quantified by ELISA Kit (Dakewe Biotech Co., Ltd., Guangzhou, China).

### 2.3. Formulations of SARS-CoV-2 RBD Trimer Vaccines and Animals

The SARS-CoV-2 RBD trimer antigen was expressed by the following literature. Briefly, the amino acid sequence was designed based on reference [17]. Maxvax (MAXVAX Bio-tech Co., Ltd., Chengdu, China) used recombinant DNA technology to transfect and express recombinant SARS-CoV-2 S protein (RBD trimer) in Chinese hamster ovary (CHO) cells. It was then purified in a denatured form by chromatography, refolded, and formulated with excipients. Female 6–8 weeks old BALB/c mice (5–6 mice per group) were vaccinated with saline, TLR7/8 agonist (compound **2**)+antigen, and mannose-TLR7/8 agonist (compound **3**)+antigen, via subcutaneous injection on days 0 and 21. Fourteen days after the final vaccination, the mice were sacrificed and their blood was collected. The RBD antigen was the receptor binding domain (RBD) of the S1 subunit of the spike transmembrane protein, which was isolated from the COVID-19 original strain, Figure S3. Each dose of vaccine contained 5 μg of antigen and 5 μg in 100μL PBS buffer; subcutaneous injection into the back of the mouse was done using a syringe.

### 2.4. Enzyme-Linked Immunosorbent Assay (ELISA)

Antigen-specific antibody titer was analyzed by ELISA using the SARS-CoV-2 RBD trimer as coating antigens. The expression and purity of the protein is described in Figure S3. We coated 96-well ELISA plates (Sangon Biotech, Shanghai, China) with 100 μL/well of 1 μg/mL antigen overnight. These wells were washed three times by PBST composed with phosphate buffered saline (PBS) containing 0.5% (w/w) of Tween 20 (Solarbio bioscience, Peking, China) and then blocked with 200 μL/well of 2% bovinse serum albumin (BSA, Aladdin, Haishang, China) for 2 h at room temperature. After blocking, the liquid was discarded; then, 100 μL sera samples (1:200,000 dilution) were added and incubated for 2 h at room temperature. After incubation, the wells were washed three times by PBST, and anti-mouse Ig Horseradish peroxidase (HRP) labeled antibody (Immunoglobulin G,IgG 1:6000 dilution, IgG1 1:15,000 dilution, IgG2a 1:1000 dilution, IgG2b 1:10,000 dilution, Thermo fisher, IgM 1:2000 dilution, Sangon Biotech, Shanghai, China) was added and incubated for 1 h. After incubation and washing five times by PBST, 100 μL of chromogenic substrate 3,3’,5,5’-tetramethylbenzidine (TMB, Biofix, Rochester, NY, USA) was added to each well and incubated for 10 min at room temperature. We added 100 μL 1 M hydrochloric acid (HCl) to each well to stop the reaction. Absorbance readings at 450 nm were read on a SpectraMax-M2 plate reader. The calculation method of sample titer is in the Supplementary Materials Section.

### 2.5. Statistical Analysis

The data from experiments were analyzed by unpaired *t*-test and one-way ANOVA with Bonferroni–Dunn post-hoc test in GraphPad Prism 8. EC50 values were calculated using a nonlinear regression model. Results were reported as mean ± standard deviation (SD). A value of *p* < 0.05 was considered statistically significant.

## 3. Results

### 3.1. Synthesis and Characterization of Compound 3

**Scheme 1:**
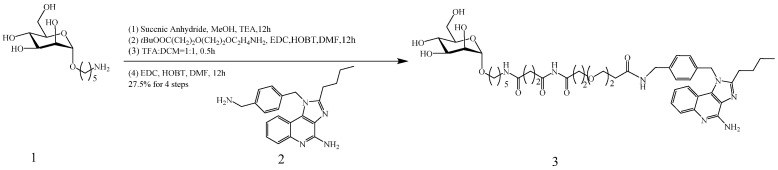
Synthesis of compound **3** (mannose and TLR7/8 agonist conjugate).

**Figure 1:**
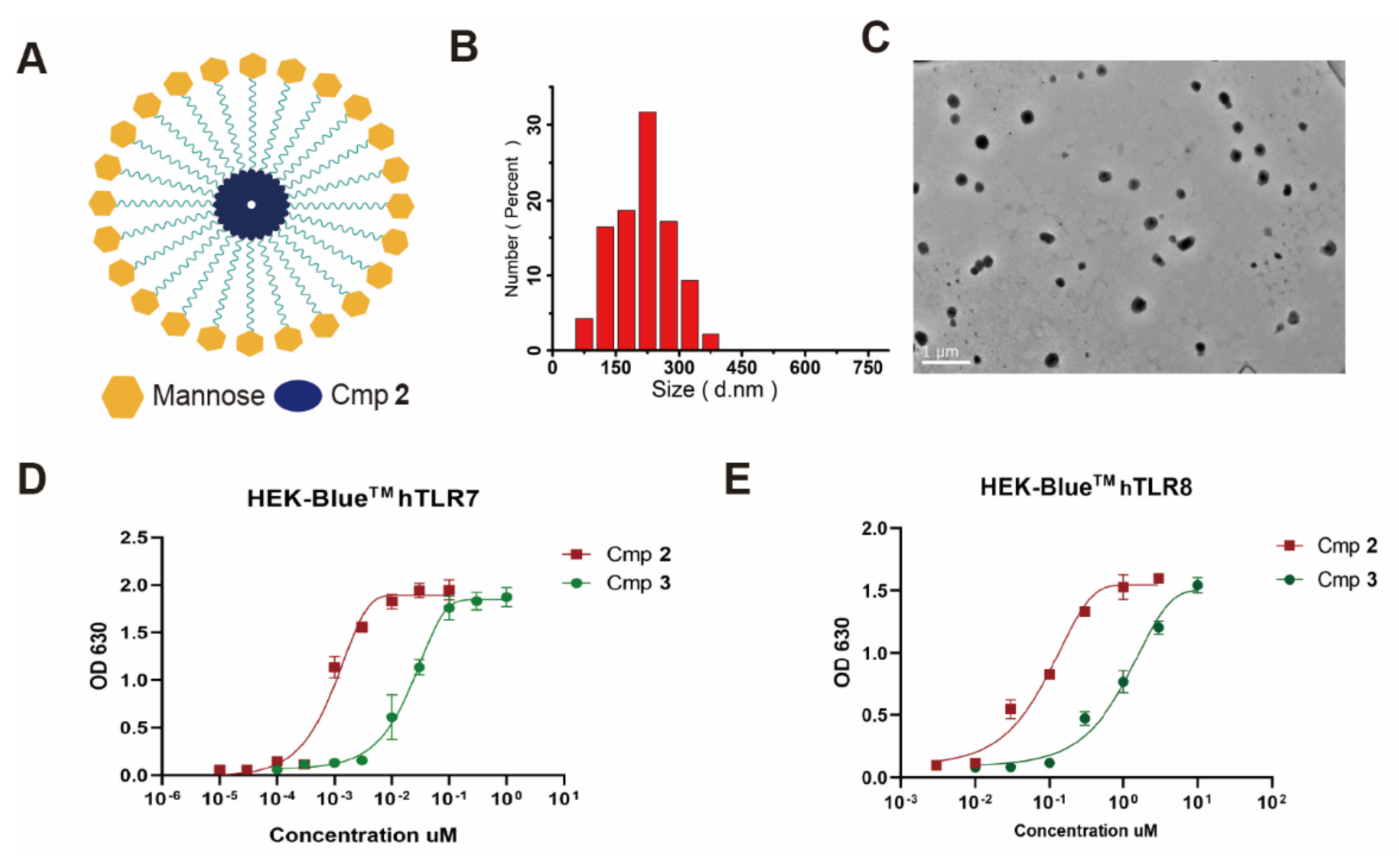
**Self-assembled compound 3 (Cmp 3) stimulated TLR7 and TLR8 signaling.** (**A**) Schematic diagram of self-assembled nanoparticles; (**B**) The nanoparticles diameter and size distribution were analyzed by Image J image software and origin 9 analysis software. (**C**) The TEM image (1.5 mg/mL) in PBS, 1% uranyl acetate solution was used to stain the sample, scale bar shows 1 μm, (**D**,**E**) HEK-Blue^TM^ hTLR7 (EC50, compound **2**: ~0.001μM, compound **3**: 0.017μM) and hTLR8 (EC50, compound **2**: 0.045μM, compound **3**: 0.6μM) cells were treated with the compound **2** and **3** for 14 h at the indicated concentrations, and the final OD values were collected at 630 nm. The data are shown as the mean ± SD (n ≥ 3).

Compound **1** was prepared according to the literature report [18]. Then, it was carboxylated by using succinic anhydride, which was coupled with a short PEG linker to distance the mannose-targeting moiety and the TLR7/8 agonist (compound **2**). The tert-butyl group was removed under acidic condition, followed by the coupling with compound **2** to afford target compound **3** as shown in Scheme 1. The detailed synthesis procedures are described in procedure S1 and the NMR data is shown in Figures S1 and S2. As shown in Figure 1A, compound **3** was expected to self-assemble to form nanoparticles with mannose on the outside and TLR7/8 agonist inside by simply dropping the DMSO solution of compound **3** to stirred PBS, followed by sonication. The particle size ranges between 50 and 400 nm, as shown in Figure 1B,C. The zeta potential of compounds has extended from 10.18 mV (compound **2**) to -27.37 mV (compound **3**), indicating a better stability behavior of compound **3** colloid (Table S1).

Then, we studied whether compound **3** still maintained its TLR7/8 activation ability. HEK-Blue^TM^ hTLR7 and hTLR8 cells express the human TLR7 and TLR8 genes, respectively. Compared with the parent compound **2**, the EC50 values for both cells (Figure 1D,E) significantly dropped, but still maintained at a low micromolar range.

### 3.2. Compound **3** Induced BMDC Maturation and Stimulate Cytokines Secretion

Mouse BMDCs exhibit the phenotype of immature DCs, displaying endocytic activity and marked expression of major histocompatibility complex (MHC) class II, intercellular adhesion molecule 1 (ICAM-1; CD54), and the integrin CD11c [19]. Previous reports indicated TLR7/8 agonists could stimulate IRF3 and IRF7, playing an important role in the polarization of mouse BMDCs [20].

**Figure 2:**
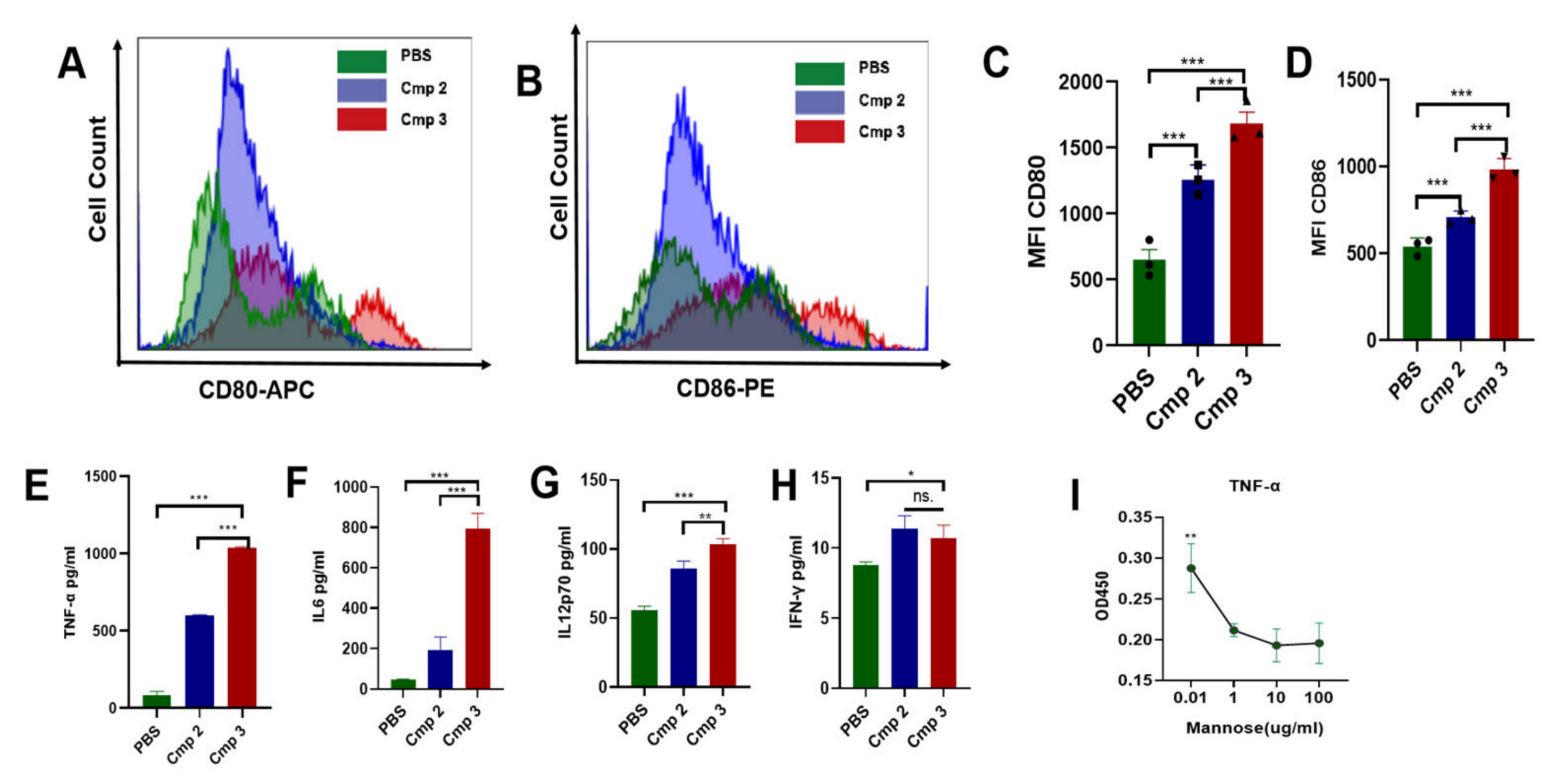
**Compound 3 could induce BMDC maturation and stimulate cytokines Secretion.** (**A**,**B**) Maturation of BMDCs was evaluated by measuring the expression of the surface molecules CD80 and CD86 by flow cytometry after 24 h of co-culture with PBS, compound **2** or **3** (5.0 µM). (**C**,**D**) The mean fluorescence intensities (MFI) of costimulatory molecules CD80 and CD86. (**E**–**H**) TNF-α, IL-6, IL12-70, and TNF-γ levels were measured from cell culture supernatants using ELISA. (**I**) TNF-α levels were quantified by ELISA from BMDC culture supernatants treated with 5.0 µM compound **3** and different concentrations of mannose. Significant differences among the groups were analyzed using one-way ANOVA with Bonferroni post-hoc test. The data are presented as the mean ± SD, where * *p* < 0.05, ** *p* < 0.01, *** *p* < 0.001, no significant(ns.).

BMDCs were isolated after 6 days of culture and incubated for a further 24 h with 5 µM of compound **2** or **3**. Expression of the costimulatory molecules CD80 and CD86 was analyzed by flow cytometry, as shown in Figure 2A–D. Compound **3** induced more expression of CD80 and CD86 than compound **2**. The secretion levels of IL-12p70, IL6, INF-γ, and TNF-α in the supernatants were quantified by ELISA kit, as shown in Figure 2E–H. The secretion of IL-12p70, IL6, and TNF-α in the compound **3** group were higher than the compound **2** group, especially the latter two. The self-assembled structure improved compound **2** activity by the targeting effect, wherein mannose binds to the mannose receptor of the DC cell. As can be seen from Figure 2I, TNF-α production can be inhibited by adding mannose in the culture medium, indicating the targeting effect of the mannose moiety in the conjugate.

### 3.3. Compound **3** as an Effective Vaccine Adjuvant for SARS-CoV2 RBD Trimer Antigen

Next, we explored its adjuvant potency for the SARS-CoV-2 RBD trimer protein. The protein mediates viral entry into host cells by first binding to a host receptor and has been proven as an effective vaccine antigen. Compound **3** was used as the adjuvant to take advantage of the mannose targeting effect and the TLR7/8 agonist adjuvant effect.

**Figure 3:**
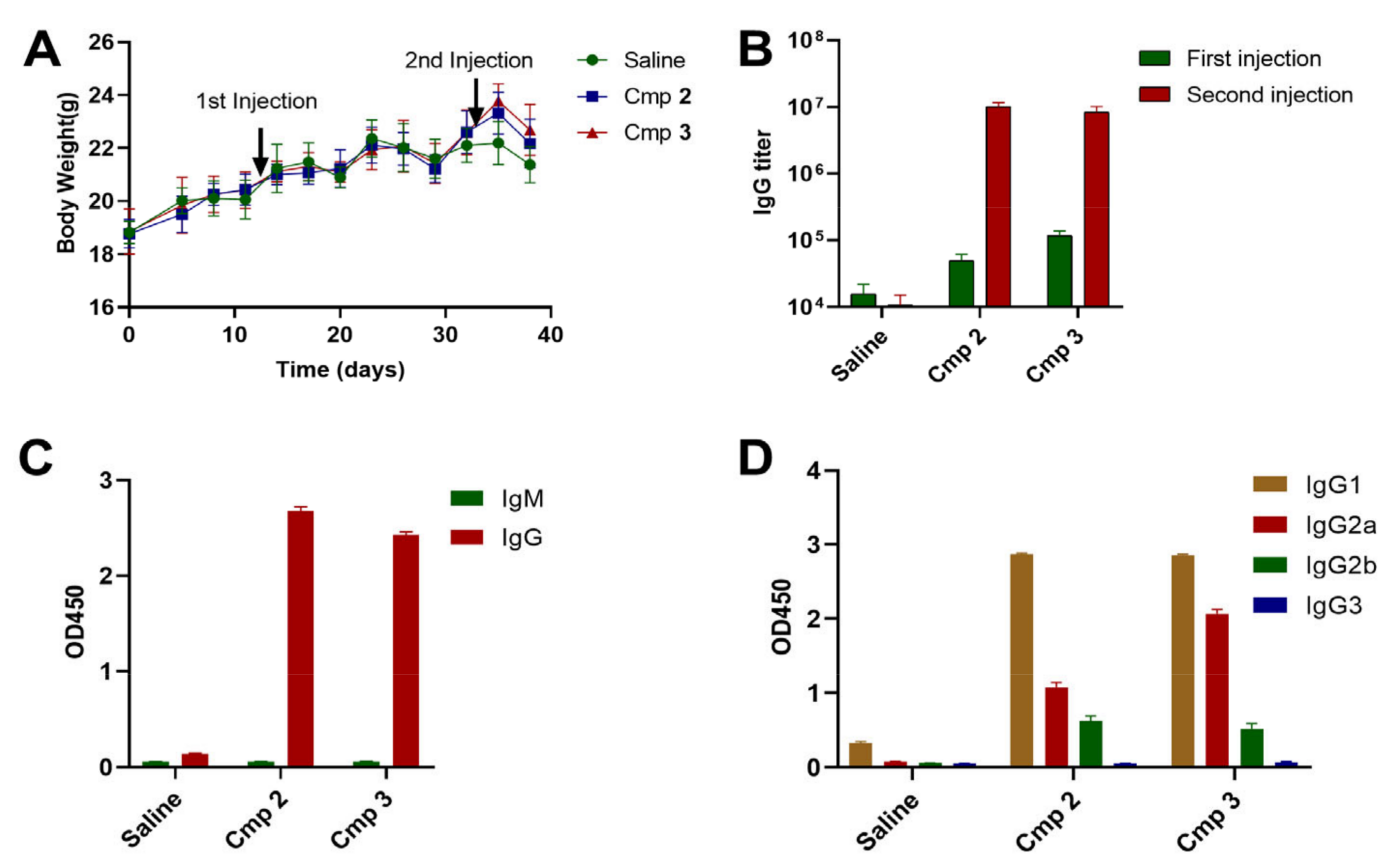
**Compound 3 as an effective vaccine adjuvant for SARS-CoV2 RBD trimer antigen.** Balb/c mice were injected subcutaneous with saline, compound **2**+antigen, and compound **3**+antigen two times at 3-week intervals. (**A**) Body weight was measured at the indicated times (n ≥ 5). (**B**) The total antibody IgG titers of different individuals’ sera derived from mice after first or second immunization. (**C**,**D**) IgM, IgG, and IgG subtypes include IgG1, IgG2a, IgG2b, and IgG3 levels against the RBD protein of the pooled sera (sera 1:200,000 dilution); all antisera were collected two weeks after the second immunization. Each data represents the total OD450 absorbance value. The data are presented as the mean ± SD (n ≥ 3).

The IgM, total IgG, IgG1, IgG2a, IG2b, and IgG3 levels were determined by ELISA. In general, a significant boosting effect was observed for both adjuvants. As shown in Figure 3B, the total IgG levels were similar for both adjuvants. The IgM levels at 1:200,000 dilution were very low compared with IgG (Figure 3C), indicating successful antibody class switching occurred rapidly after activation of mature naïve B cells. Compound **3** induced more IgG2a than compound **2** (Figure 3D), but the mechanism behind this phenomenon is not very clear.

## 4. Discussion

**Figure 4:**
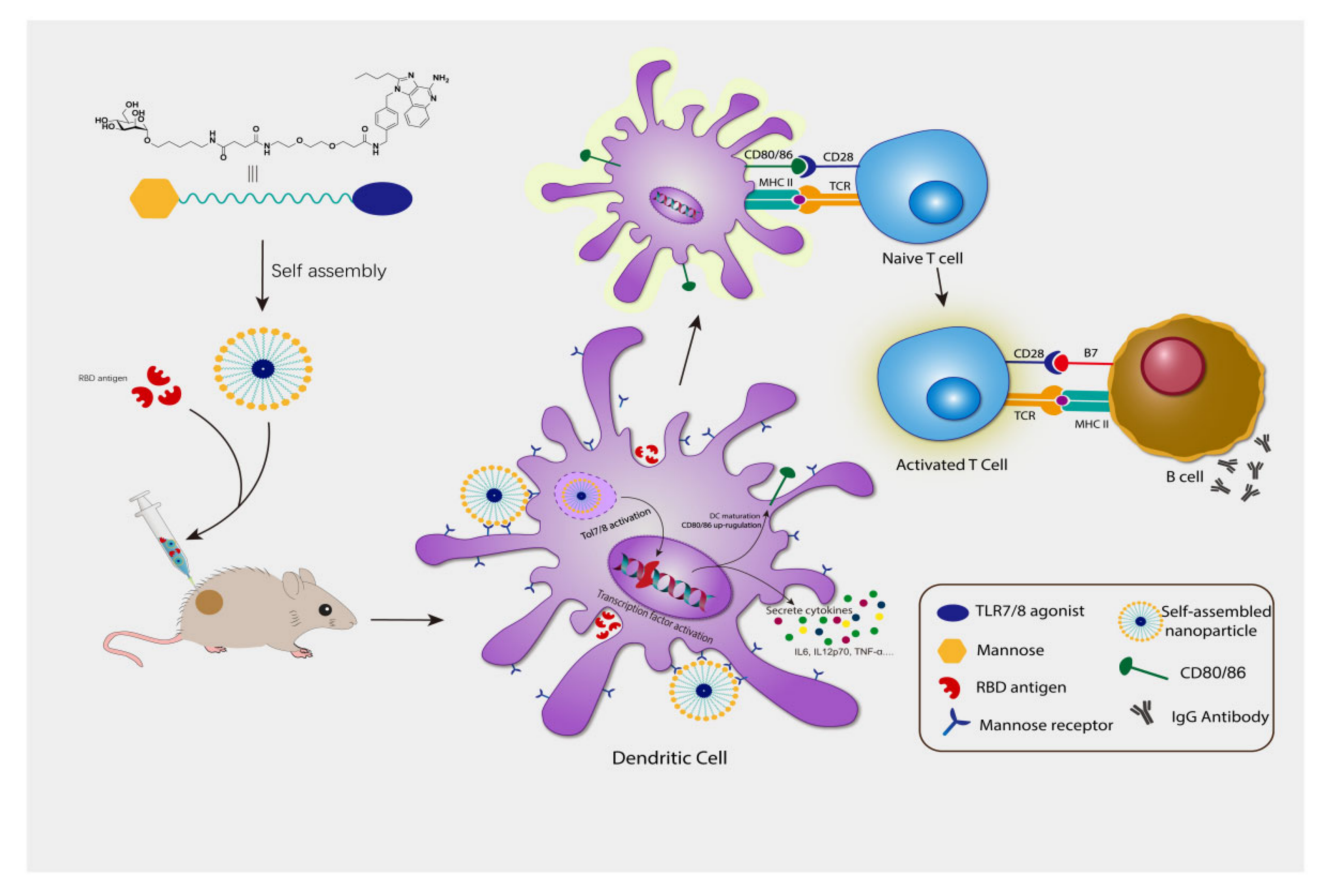
Concept of mannose-TLR7/8 agonist conjugate nanoparticle fabrication and immune cells stimulation.

Imidazoquinoline derivatives (IMDs) are effective TLR7/8 agonists. Due to their high efficacy and simplicity, they have been extensively studied in vaccines and anti-tumor research. To address their poor pharmacokinetic properties and toxicities associated with systemic administration, various strategies relying on bio-conjugation and nanoparticles were explored, which has been thoroughly reviewed elsewhere [1,2]. However, those approaches were technically nontrivial. For example, efficiency of the reported polymeric preparation of TLR7/8 conjugates was found to be low in our study: only ~20% of the reactive sites of a polymer can be conjugated (unpublished data). Here, a new approach was proposed by coupling a TLR7/8 agonist with mannose through a short PEG linker. The synthesis was relatively easy, and the generated compound could self-assemble to form particles targeting C-type lectins of the DC surface via the surface polymeric mannoses. As shown in Figure 4, the prepared compound **3** was dissolved in DMSO and then added dropwise into stirred PBS, followed by sonication. Compound **3** could self-assemble to form nanoparticles. Although there is no evidence, the particles most likely have hydrophilic mannose on the outside and hydrophobic TLR7/8 agonist on the inside.

In vitro experiments demonstrated that the nanoparticles induced more CD80 and CD86 expression than the unmodified compound **2**, indicating compound **3** is a more potent adjuvant in activating DCs. CD80 and CD86 are up-regulated on mature DCs and provide co-stimulation to T cells for their activation and survival via ligation with CD28. Blocking antibodies targeting those markers weakened intercellular interactions between the DC and T cell, and dampened T-cell activation, highlighting the amplificatory roles of CD80/86 in regulating DC:T-cell interactions and T-cell functional activation [21].

Previous reports indicated TLR7/8 agonists can efficiently polarize mouse BMDCs [14]. Mouse BMDCs were treated with the prepared nanoparticles and the secreted cytokines were analyzed. A significant difference was observed for interleukin 6 (IL-6). Compound **3** induced a more than 18-fold increase than the blank control and 4-fold than compound **2**. IL-6 has pleiotropic action on different cell types of the immune system, which acts as both a pro-inflammatory cytokine and an anti-inflammatory cytokine [22]. It seems IL-6-/- PMDC live longer, indicating IL-6 may promote the PMDC to mature [23,24]. IL-6 is essential for optimal and complete in vivo response of CD8+ T cells [25]. It promotes the differentiation of CD8+ T cells into cytotoxic T cells [26]. IL-6 also promotes specific differentiation of naïve CD4+ T cells, thus performing an important function in the linking of innate to acquired immune response [27]. Compound **3** also induced a higher amount of IL-12p70 and TNF-α than compound **2**, as an example, the latter of which can be partially suppressed by adding mannose into the culture medium indicating the targeting effect. It seems both compounds did not impact the IFN-γ production. The data indicated compound **3** is a potent mouse BMDC activator.

Next, we explored compound **3**′s potency as a SARS-CoV-2 vaccine adjuvant by in vivo experiments. By using either compound **2** or **3**, antibody class can be successfully switched from IgM to IgG. Compound **3** induced more IgG2a than compound **2** (Figure 3D). Further study is required to investigate the mechanism of higher IgG2a. Antibodies can not only neutralize the corresponding antigen, but more importantly, can mediate the host effector to facilitate the removal of a pathogen from a host. The Fc portion of IgG2a antibodies interacts with FcRIV to stimulate the antibody-dependent cell-mediated cytotoxicity and opsonophagocytosis by macrophages. The Fc portion of IgG1 antibodies mediates a lower-affinity interaction with activatory Fc receptors and does not stimulate Fc receptor-mediated immune responses as effectively. For these reasons, among IgG subclasses, IgG2a exhibits the best Fc receptor activatory-to-inhibitory ratio of all IgG isotypes [28], making it the isotype predicted to have the greatest ability to activate Fc receptor-mediated host effector responses [29]. The trend has been proven by in vivo study: IgG2a is more efficient at clearing influenza, Ebola, and yellow fever virus infections than IgG1 [30,31,32,33,34,35]. These data indicate that the current vaccine may more efficiently clear SARS-CoV-2, since more IgG2a is generated.

## 5. Conclusions

The development of effective and safe vaccines is the key to fight against pathogens like SARS-CoV-2. However, vaccine efficacy varies and is affected by the waning of antibodies and the emergence of mutations. Currently, most vaccines are based on traditional adjuvants like aluminum, QS21, etc. Novel adjuvants are in desperate need for prolonged protection. We prepared a conjugate to take advantage of the mannose targeting effect and the strong adjuvant ability of TLR7/8 agonists and studied its adjuvant ability. The results indicated that the conjugate is superior in activating BMDCs and generated a high level of TNF-α and IL6. Coinciding with the cytokine result, it also generated more IgG2a, indicating compound **2**′s high potential as a vaccine adjuvant. However, it should be noted that other studies are required to reveal the mechanism and underlying implications of such a phenomenon. For example, how does the secretion of more TNF-α and IL6 impact the cellular response? Whether more IgG2a can provide additional immunity against SARS-CoV-2 has not been studied due to biosafety concerns. Further research may focus on studying the impact of the linker between mannose and TLR7/8 adjuvants, simplifying the synthesis, studying the mechanism of more IgG2a secretion, and the reactivity comparison with other adjuvants.

## Data Availability

All data generated or analyzed during this study are included in this published article and its supplementary information files.

## Supplementary Materials

The following supporting information can be downloaded at: https://www.mdpi.com/article/10.3390/polym14245466/s1, Procedure S1: Synthesis of compound **3**; Figures S1 and S2: 1D ^1^H and HSQC NMR spectra of compound **3**.
