# Supplementary material for: Self-Assembled TLR7/8 Agonist-Mannose Conjugate as An Effective Vaccine Adjuvant for SARS-CoV-2 RBD Trimer
Source: Polymers (Basel). 2022 Dec 13;14(24):5466. doi: 10.3390/polym14245466 (PMC9785909; doi:10.3390/polym14245466)
Supplement: Supplementary file 1 [file polymers-14-05466-s001.zip › polymers-1939822-supplementary.pdf]

Self-assembled TLR7/8 agonist-mannose conjugate as an effective vaccine adjuvant for SARS-CoV-2 RBD trimer

Changcai Teng [1], Xiongyan Meng [1], Yeqin Hu [2], Hongzhao Mao [1], Huiting Li [1], Jing Yang [1], Tiantian Sun [1], Shuai Meng [1] and Chengli Zong [1,*]

[1] Key Laboratory of Tropical Biological Resources of Ministry of Education, School of Pharmaceutical Sciences, Hainan University, Haikou 570228, China
[2] MAXVAX Bio-Tech Co., Ltd., Chengdu 610200, China
* Correspondence: chengli.zong@hainanu.edu.cn

Table of Contents

PdI and ZP of these two compounds	Table S1
Synthesis of compound **3**	Procedure S1
^{1}H NMR spectra of compound **3**	Figure S1
HSQC spectra of compound **3**	Figure S2
SDS-PAGE electrophoresis and Western Blot result of RBD trimer	Figure S3

Table S1 The polydispersity index and zeta potential of these two compounds.

Sample name	Zetazeta potential (mV, mean±SD)	Polydispersity index (mean±SD)
Cmp 2	10.18±2.19	0.47±0.04
Cmp 3	-27.37±2.98	0.43±0.02

These data was detect by Zetasizer Nano ZS90, particle size analyzer (Zetasizer Nano ZS90, Malvern Panalytical, city, country).

Procedure S1. Synthesis of compound **3**

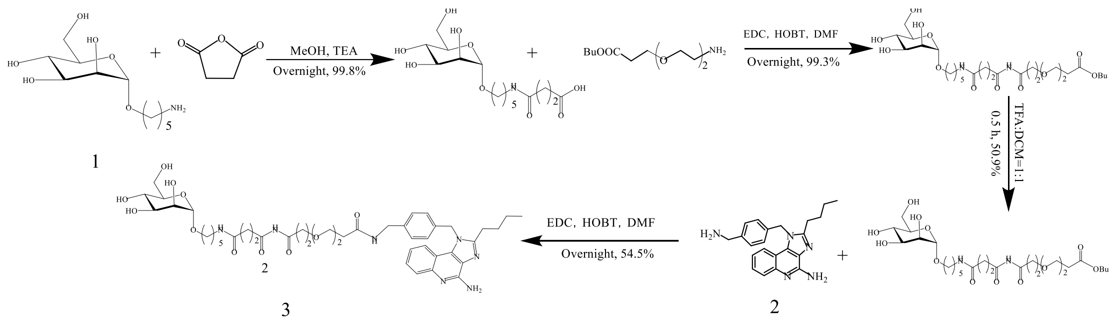

N1-(2-(2-(3-((4-((4-amino-2-butyl-1H-imidazo[4,5-c]quinolin-1-yl)methyl)benzyl)amino)-3-oxopropoxy)ethoxy)ethyl)-N4-(5-(((2S,3S,4S,5S,6R)-3,4,5-trihydroxy-6-(hydroxymethyl)tetrahydro-2H-pyran-2-yl)oxy)pentyl)succinamide (3)

A solution of **1** (800mg) in H_2O/MeOH (1:1, 8mL) was stirred with a tablespoon Pd/C under H_2 atmosphere at room temperature overnight. After the disappearance of starting material monitored by TLC, the reaction mixture was filtered through a 0.22um Nylon microporous membrane. The solvent was removed under reduced pressure. Succinic anhydride (2.2 equiv, 83mg) was added to a solution of the residue (100mg) in MeOH (3mL), then 105μL TEA was added to the mixture, The reaction was stirred at room temperature overnight. The product was purified by P2 chromatography. Then, the intermediate (60mg) and $BuOOC(CH_2)_2O(CH_2)_2OC_2H_4NH_2$ (76.6ul) were dissolved in DMF (3mL), followed by the addition of EDC(47.2mg), HOBT(33.3mg). After 12 hours, the product was purified by P2 chromatography. The product was dissolved in DCM (1mL), and then mixed with the same volume of TFA, The reaction was stirred at room temperature for 30 min. The product was purified by P2 chromatography.

The intermediate was coupled with compound **2** in DMF (3mL) by using EDC(47.2mg), and HOBT(33.3mg). The reaction was stirred at room temperature for 12 hours. The

product was separated and purified by silica gel. The yield is 27.5% for 4 steps.^{1}H NMR (500 MHz, MeOD) δ 7.70 (d, *J* = 8.3 Hz, 1H, H-28), 7.56 (d, *J* = 8.4 Hz, 1H, H-25), 7.34 – 7.28 (m, 1H, H-26), 7.17 (d, *J* = 7.9 Hz, 2H, H-22), 7.00 (t, *J* = 7.5 Hz, 1H, H-27), 6.91 (d, J = 7.9 Hz, 2H, H-21). 5.76 (s, 2H, H-23), 4.63 (s, 1H, H-1), 4.25 (s, 2H, H-20). 3.72 (dd, *J* = 11.7, 2.4 Hz, 1H, H-6a), 3.68 (dt, *J* = 2.9, 1.3 Hz, 1H, H-2), 3.65 – 3.57 (m, 5H, H-6b,18,7a, 3), 3.52 (t, J = 9.4 Hz, 1H, H-4), 3.43 (dd, J = 5.8, 3.5 Hz, 3H, H-16,5), 3.39 – 3.34 (m, 2H, H-17), 3.32 – 3.24 (m, 3H, H-7b,15), 3.15 (t, J = 5.5 Hz, 2H, H-14), 3.03 (t, J = 6.9 Hz, 2H, H-11), 2.86 (t, J = 7.7 Hz, 2H, H-24). 2.40 – 2.30 (m, 6H, H19, 12&13), 1.68 (p, J = 7.7 Hz, 2H, H-29), 1.47 (dq, *J* = 12.9, 6.5 Hz, 2H, H-8), 1.41 – 1.30 (m, 3H, H-10,30), 1.27 (dt, *J* = 15.1, 8.3 Hz, 2H, H-9), 0.85 – 0.80 (m, 3H, H-31).13C NMR (126 MHz, MeOD) δ 130.11, 129.40, 127.84, 127.69, 127.14, 124.32, 122.45, 116.66, 102.38, 75.46, 73.48, 73.10, 72.08, 71.38, 69.51, 69.13, 63.70, 49.69, 44.41, 41.17, 38.47, 33.20, 31.74, 31.00, 28.69, 25.53, 24.27, 14.95.

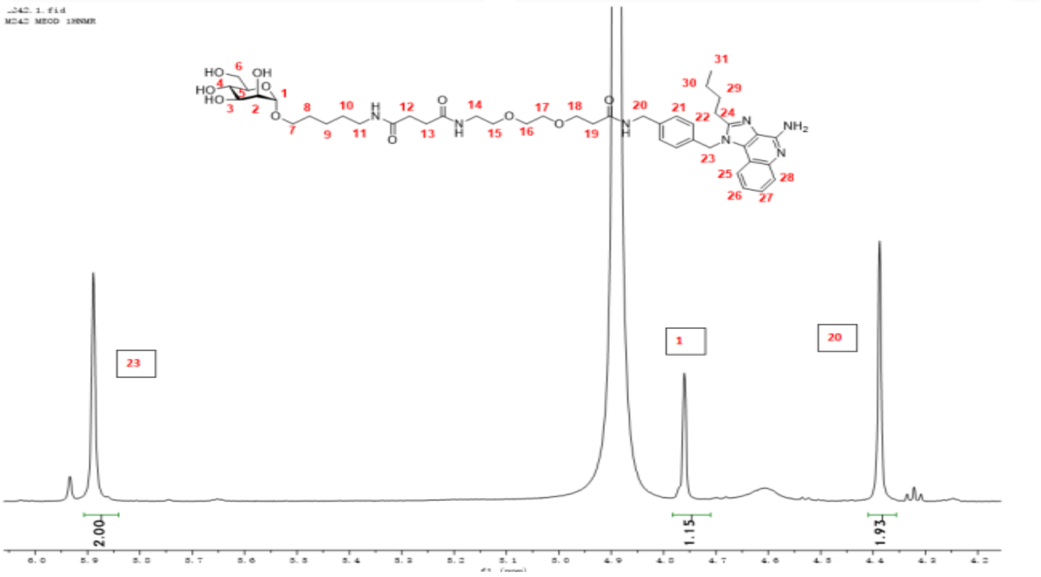

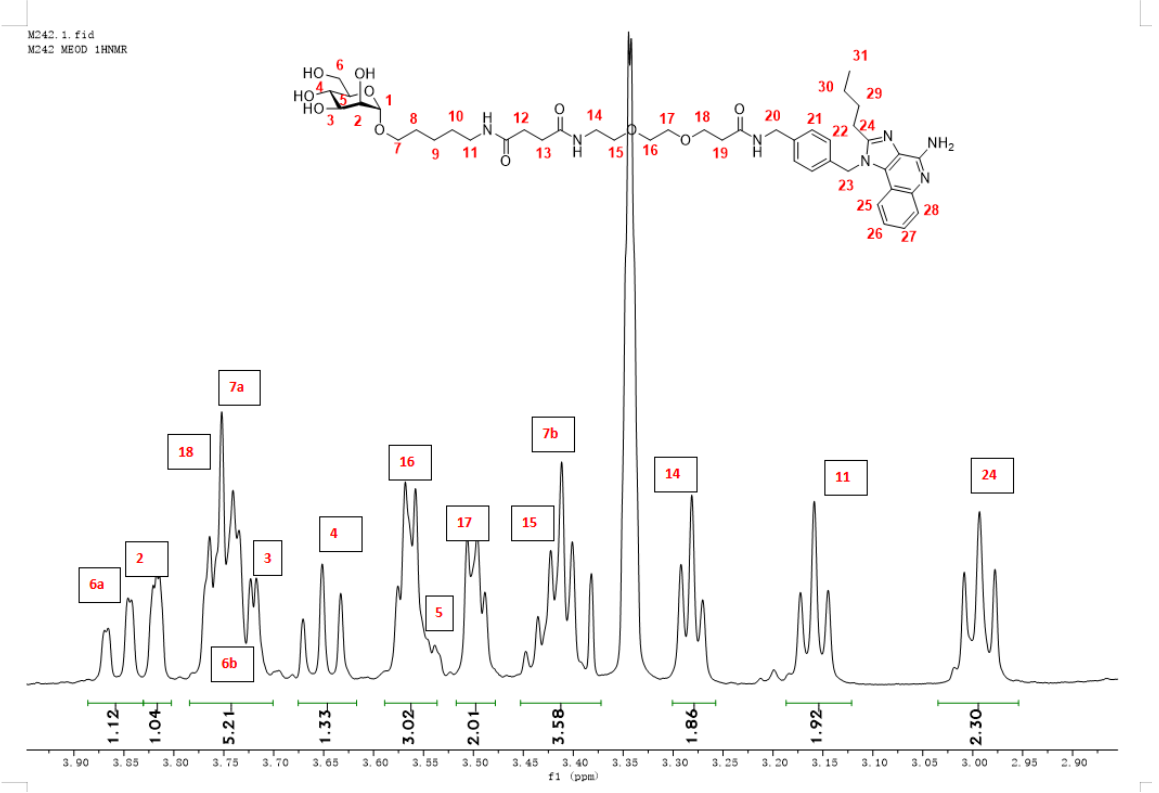

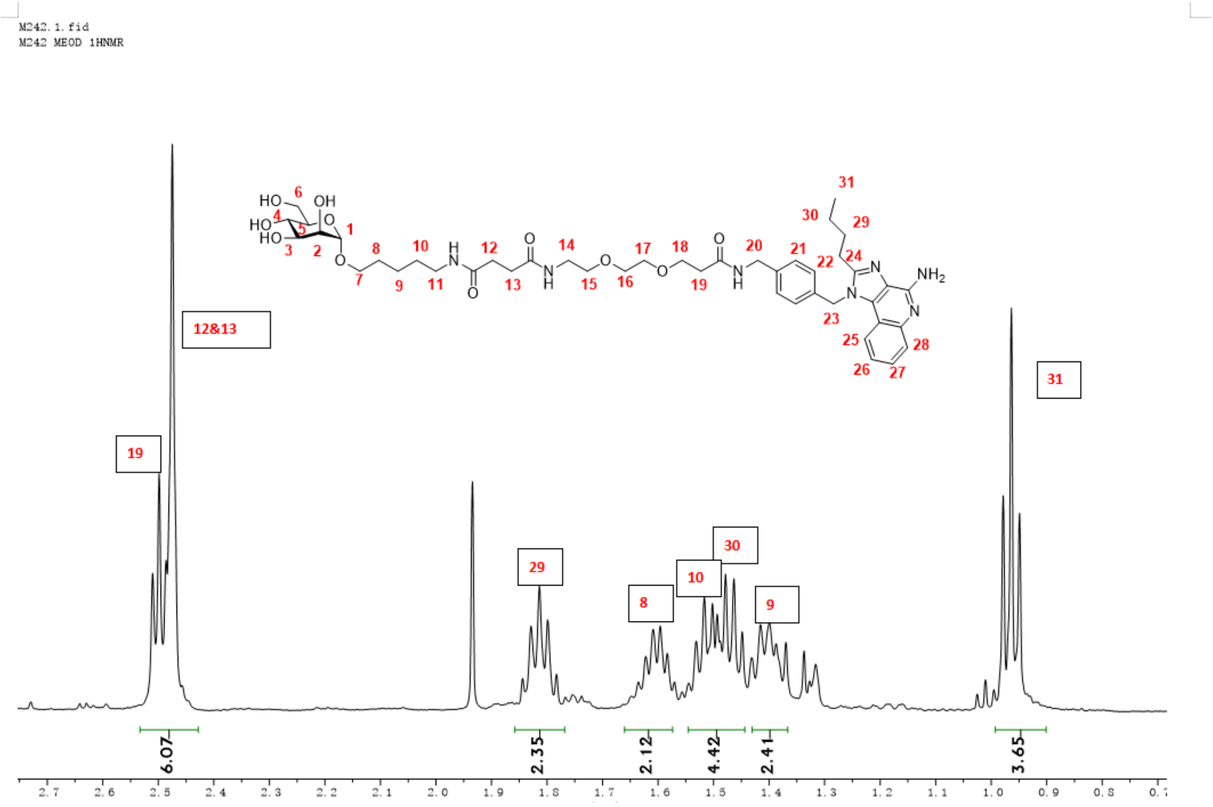

Figure S1. 1H NMR spectra of compound **3.**

The numbering numbered carbons that have at least one proton attached. The numbering starts from sugar, to facilitate the NMR interpretation.

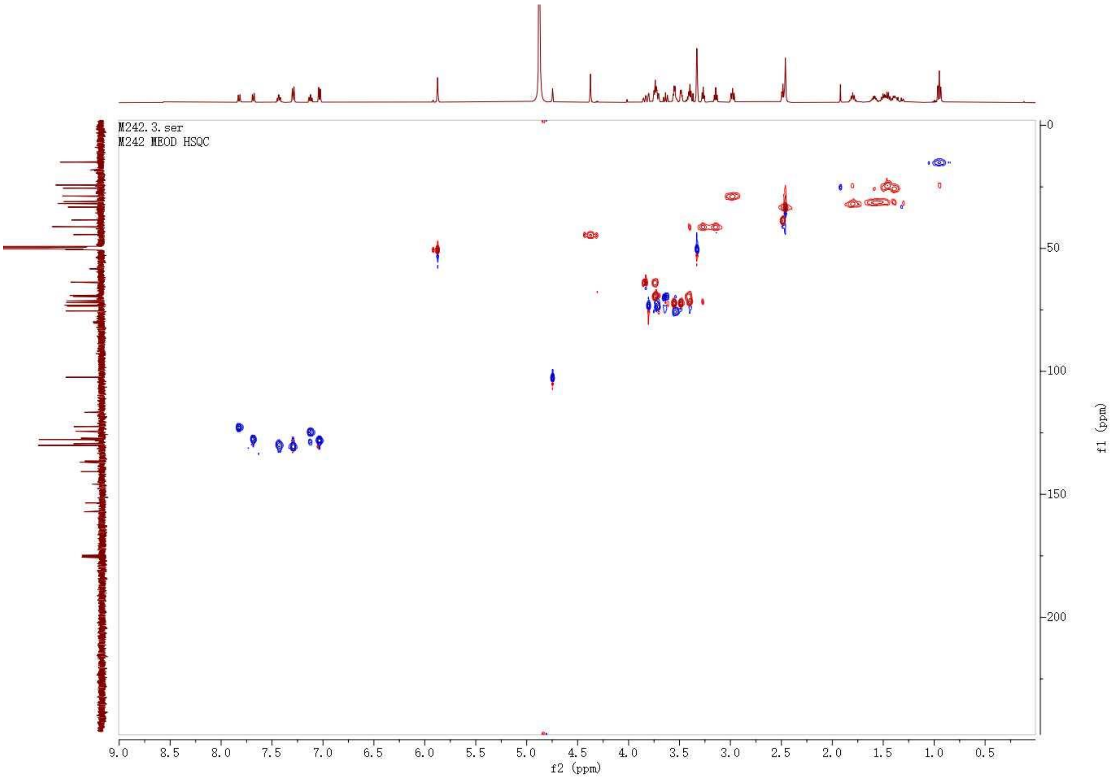

Figure S2 HSQC spectra of compound **3.**

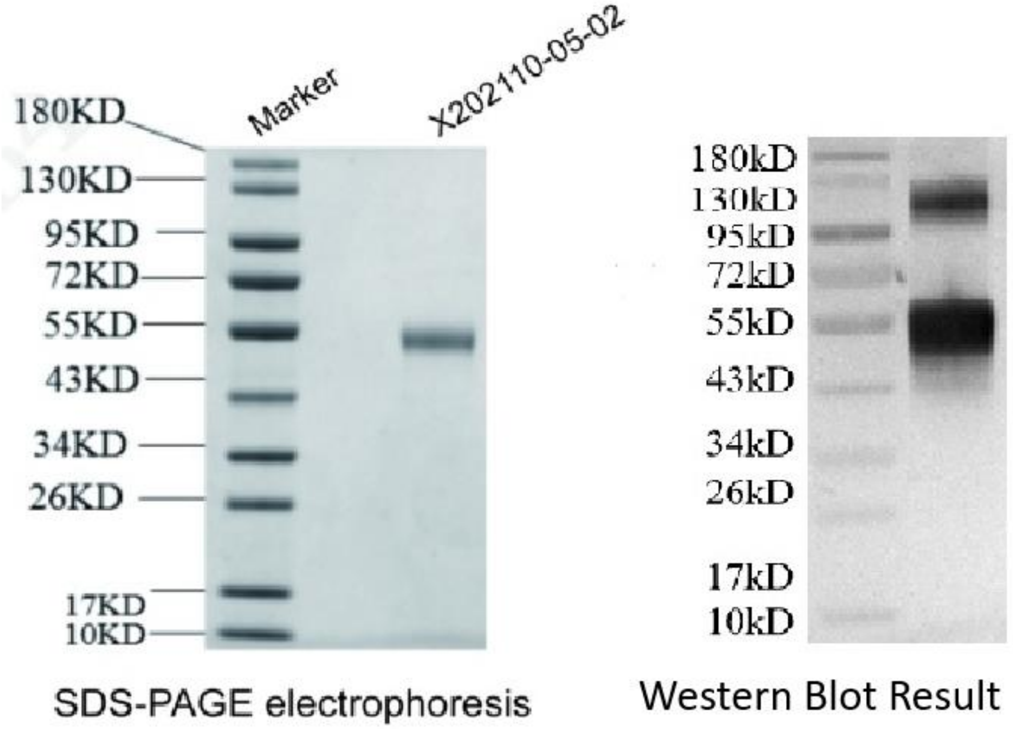

Figure S3 SDS-PAGE electrophoresis and Western Blot result of RBD trimer

The SARS-CoV-2 RBD trimer antigen was expressed by following literature. The amino acid sequence was designed based on reference [17]. Briefly, The gene encoding SARS-CoV-2-RBD-trimer (GenBank accession number: EPI_ISL_402119, S protein residues 918-966, a 22-amino-acid linker LVPRGSGGSGGSGGLEVL FQGP, 1163-1203, and 319-537) was cloned into the pKS001 expression vector (Quacell Biotechnology) using the HindIII and EcoRI restriction sites with an N-terminal signal peptide. CHO cells were electroporated with the expression vector containing coding sequences to generate a stable cell line. Transfected cells were subjected to various selection/amplification methods and single cellular clones were isolated and evaluated. Best clone was chosen according to high expression, production in fed batch and the quality profiles. Selected clone was subjected to expression process. The expressed trimer antigen was then purified in a denatured form by chromatography, refolded and formulated with excipients.Above are representative SDS-PAGE electrophoresis and Western Blot result of purified recombinant SARS-CoV-2 S protein from CHO cells (.SARS-CoV-2/2019-nCoV Spike Antibody,Rabbit PAd,Antigen Affinity Purified，Sino Biological /40591-T62 sample loading 0.3 μg).
